# Processing Aspectual Agreement in a Language with Limited Morphological Inflection by Second Language Learners: An ERP Study of Mandarin Chinese

**DOI:** 10.3390/brainsci12050524

**Published:** 2022-04-21

**Authors:** Yuxin Hao, Xun Duan, Qiuyue Yan

**Affiliations:** 1 Institute of Chinese Language and Culture Education, Huaqiao University, Xiamen 361021, China; 2 College of Chinese Language and Culture, Huaqiao University, Xiamen 361021, China; 19014041006@stu.hqu.edu.cn (X.D.); 18014041031@stu.hqu.edu.cn (Q.Y.)

**Keywords:** Mandarin Chinese, aspect, second language learners, processing, ERP

## Abstract

Previous studies on the neural cognitive mechanisms of aspectual processing in second language (L2) learners have focused on Indo-European languages with rich inflectional morphology. These languages have aspects which are equipped with inflected verb forms combined with auxiliary or modal verbs. Meanwhile, little attention has been paid to Mandarin Chinese, which has limited morphological inflection, and its aspect is equipped with aspectual particles (e.g., le, zhe, guo). The present study explores the neurocognitive mechanism of Mandarin Chinese aspect processing among two groups of late Mandarin Chinese proficient learners with Thai (with Mandarin Chinese-like aspect markers) and Indonesian (lack of Mandarin Chinese-like aspect markers) as their first language (L1). We measured event-related potentials (ERPs) time locked to the aspect marker *le* in two different conditions (the aspect violation sentences and the correct sentences). A triphasic ELAN-LAN-P600 effect was produced by the Mandarin Chinese native speakers. However, there was no ELAN and LAN in Indonesian native speakers and Thai native speakers, except a 300–500 ms negativity widely distributed in the right hemisphere and P600-like effect. This suggests that both groups of Mandarin Chinese learners cannot reach the same level as Mandarin Chinese native speakers to process Mandarin Chinese aspect information, probably due to the complex feature of Mandarin Chinese aspect maker, the participants’ L2 proficiency and age of L2 acquisition.

## 1. Introduction

Tense and aspect are important means for human language to encode time information [1]. “Tense” refers to the time when an action occurs with the current time as the reference, such as the present and past [2]. “Aspect” involves different ways of viewing the temporal characteristics of action states [3]. Among them, the grammatical category aspect (also known as viewpoint aspect) refers to a state, or a series of progressive states, that a speaker observes at a given time, or from a series of time viewpoints [4,5]. Generally speaking, the viewpoint aspect is divided into perfect aspect and progressive aspect. The perfect aspect entails looking at a situation from the outside, presenting an indivisible whole, an achievement or a complete event. The progressive aspect entails looking at a situation from the inside, and the presented situation is an ongoing process, usually continuous, and implying transience, dynamics, and willingness [6]. In Indo-European languages, the aspect is usually expressed by verbs’ morphological inflections, as in Spanish, for example, “Está (he) comiendo (is eating)”. However, in addition to the morphological inflections of verbs, the expression of “aspect” in some languages (e.g., English) also need the help of auxiliaries. Taking sentence (1) as an example, while the perfective aspect is expressed by “has + v-ed”, indicating that the action has been completed, the progressive aspect is expressed by “is + v-ing”, indicating that the action is in progress.

a. So far, John has learned his courses b. Right now, John is learning his courses
(1)

Chinese aspect markers are used as post-verb markers or pre-verb markers to represent verb aspects [7]. They are morphologically similar to the inflectional suffixes of most Indo-European verbs, and they are attached after verbs [8,9,10]. For example, the aspect marker le in (2a) indicates that the action is complete, and zhe in (2b) indicates that the action is in progress.

a. 安娜   看 了    比赛 Anna  kan *le*     bisai Anna  watch-*le*  bisai Anna  watched  the game b. 安娜     看 着       比赛 Anna   kan *zhe*      bisai Anna  watch-*zhe*     bisai Anna  is watching  the game(2)

Previous studies using ERPs have investigated the processing of time reference words (time nouns or time adverbs) and aspect violations. These studies, however, mainly focused on native speakers or second-language speakers of Indo-European languages. Among the few existing studies on this topic in Chinese, almost all of them focused on Chinese native speakers. The purpose of this study is to take Mandarin Chinese native speakers as a reference and use an event-related potential (ERP) technique to explore the neurocognitive mechanism of Mandarin Chinese aspect markers processed by L2 learners with different L1 backgrounds, and to explore the factors affecting the processing of Mandarin Chinese aspect among L2 learners.

### 1.1. ERP Studies on the Aspect and Tense

Over the past two decades, ERP research on the processing mechanism of time information has accumulated. In Indo-European languages, if there are multiple time reference markers in a sentence, these time markers must have a formal agreement. For example, in an English sentence, the verb’s tense (e.g., past tense, present tense) and aspect (e.g., perfect aspect, progressive aspect) must be consistent with the time meaning indicated by the time adverb (past, present, future) (as shown in (3)).

a. Right now, Sophie *is swimming* in the pool. b. * Right now, Sophie *swims* in the pool. (3)

The * means that the sentence is incorrect. This symbol appears below also expresses this meaning.

In (3a), “is swimming” is the verb form of the progressive aspect, indicating that the action is in progress. This is consistent with the time adverb “right now” pointing to the present. On the contrary, the temporal meaning of “swims” and “right now” in (3b) is inconsistent, resulting in temporal and aspectual violations.

Previous ERP studies have focused on tense and aspect violations in native Indo-European speakers [11,12,13,14]. They found that verbs with aspect violation generally elicited a LAN (or a LAN-like component) and a P600. While some researchers interpreted the LAN-P600 pattern as a morphosyntax processing of aspect violation (e.g., Steinhauer and Ullman [12], others believed that aspect processing is neither semantic processing, nor morphosyntax processing alone (e.g., Monique et al. [14]).

Zhang and Zhang [15] conducted an ERP study on Mandarin Chinese grammatical aspect for Chinese native speakers, as shown in sentence (4). The results showed that in the time window of 200–400 ms, the aspect violation sentence elicited a posterior and left central negativity, followed by a P600. The authors argued that this negativity (200–400 ms) may reflect the failure of matching the aspect marker with the time adverb in the sentence, or the detection errors of aspect violation. The subsequent P600, however, represented syntactic repair or monitoring and processing of violations caused by aspect inconsistency. The above findings were partially repeated in a follow-up study, in which the aspect violation between the Mandarin Chinese aspect marker “*guo*” and time noun led to a pronounced P600 [10].

* 苏君   正在      预备   了     水果     和   甜点 * Sujun  zhengzai  yubei   *le*  shuiguo  he  tiandian * Sujun  zhengzai  prepare-*le*  fruits     and  dessert * Sujun  is preparing      *le*   fruits    and  dessert * Sujun  zhengzai (PROG, ‘ongoing’) prepare *le* (PERF) fruit and cookies. (4)

In sum, most studies on L1 tense and aspect processing have focused on Indo-European languages, with less attention on Mandarin Chinese. LAN (or LAN-like components) and P600 were found in most of the ERP studies concerning tense and aspect processing in Indo-European. Moreover, early negativity and P600 have been found in ERP research on temporal information processing in Mandarin Chinese L1. This implies that the aspect processing in both Indo-European languages and Chinese could be related to syntactic processing.

In second language processing, researchers usually utilize native speakers as a reference to illustrate the potentially different processing mechanisms underlying native speakers and second language learners, by comparing second language learners with native speakers.

Tokowicz and Macwhinney [16] investigated the role of explicit and implicit processes in second language sentence processing among native Spanish speakers and Spanish learners with native English backgrounds. They divided stimulus materials into three types (as shown in (5)): (a) violation in tense and aspect markers (similar L1 and L2 structures); (b) violation in “number” agreement (L1 and L2 usage rules were different); (c) violation in “gender” agreement (grammatical items that were present in L1, but not L2). The ERP results showed that tense and aspect violation induced a P600-like effect. This suggests that L2 learners were more sensitive to the violation of similar structures in L1 and L2, but not to the violation of different structures in L1 and L2. Moreover, even L2 learners in the early learning stage could implicitly process some L2 grammar information. However, this processing mechanism depended on the similarity between L1 and L2.

a. Su   abuela       *cocinando/cocina   muy  bien His  grandmother     *cooking/cooks   very  well “His grandmother *cooking/cooks very well” b. * El/Los               niños    están  jugando * The-SING/the-PL    boys     are    playing “*The-SING/the-PL boys are playing” c. Ellos   fueron   a       *un/una       fiesta they    went    to   *a-MASC/a-FEM   party “They went to *a-MASC/a-FEM party”(5)

Julia and Harald [17] used ERP to study the neural correlates of generating complex morphological lexicon in late high-level bilinguals. They found that the past tense of regular verbs elicited more frontal-central negativity than the past tense of irregular verbs, but there was no difference in the present tense conditions. This was similar to the patterns observed in the native speakers. This study suggested that high-level bilinguals could reach a processing level similar to that of native speakers. Li et al. [18] explored whether the existence of tense in a specific language would affect the way participants processed time series events. L2 English learners with Chinese backgrounds and English native speakers judged the sentences with the modifier beginning with the connective “after” (“after + had v-ed,… V-ed…”), and the reference time expressed by the verbs of these sentences either agreed or disagreed with the main sentence. The ERP results showed that native English speakers had an N400 effect on verb violations in clauses, while L2 learners did not have an N400 effect. The absence of N400 indicated that it was difficult for L2 learners to determine the event information conveyed by the tense on the time axis. This may have been caused by the differences in expressing the time information between Chinese and English.

To summarize, these results suggest that the similarity between L1 and L2, and L2 proficiency are important factors that could influence the underlying mechanism. In addition, there has been less research on L2 processing than on L1 processing, and most of the existing studies on L2 aspect/tense processing have been mostly on Indo-European languages.

### 1.2. Comparison of Temporal Information Coding in Three Languages

As a morphologically impoverished language, Mandarin Chinese lacks the rich morphological inflections borne in Indo-European languages, and the primary means of expressing grammatical categories in Chinese are word order and functional words. Chinese also lacks grammatical forms for syntactic categories, such as number, gender, and tense [19]. However, Mandarin Chinese has a rich aspect system and several aspect markers with different functions to encode aspect information [20]. Time adverbs, time nouns, and aspect markers combined with verbs can be used to express time information in Chinese [21]. The auxiliary words “*le*” and “*zhe*” attached to the verb serve as aspect markers to indicate the completion and progress of the action, respectively [21,22]. The combination of time adverbs and aspect markers in Chinese is a way of expressing aspect in Chinese, and its expression form is shown in (6). The time adverb “*yijing*” is usually combined with the aspect marker “*le*”, and the time adverb “*zhengzai*” is often combined with the aspect marker “*zhe*”.

a. 妈妈     已经    打扫   了   房间 Mama   *yijing*   dasao   *le*   fangjian Mother  already  clean   *le*  the room Mother has cleaned the room b. 妈妈     正在     打扫     着    房间 Mama   *zhengzai*   dasao   *zhe*   fangjian Mother  zhengzai  cleaning  *zhe*  the room Mother is cleaning the room(6)

Both Thai and Mandarin Chinese lack morphological inflections, and the aspect can be expressed through aspect markers. The Mandarin Chinese aspect marker “*le*” indicates the completion of the action, and can be used with the time adverb “yijing” to indicate the past time category in simple sentence. The Thai word “แล้ว” partly corresponds to the Mandarin Chinese aspect marker “*le*”. It indicates an action’s completion, and needs to be used with a time adverb indicating the past [23]. As such, the connotations of the Thai word “แล้ว” and the Mandarin Chinese aspect mark “*le*” are consistent in expressing the aspect meaning. An example is shown in (7). In addition to expressing the completion of an action, “*le*” has other meanings in modern Chinese, such as the modal particle “*le*” [24], which does not fully correspond to “ แล้ว” in Thai. In short, the usage of “*le*” in Chinese is much more complicated than “ แล้ว” in Thai.

Thai:          คุณพ่อ    แล้ว       ชมเชย    แล้ว  พี่ชาย     และ    พี่สาว Chinese:       爸爸   已经     表扬    了  哥哥      和     姐姐 English word:  Dad   already   praise   *le*  brother   and  elder sister English: Dad has praised her brother and elder sister(7)

The Indonesian language also lacks morphological inflections. The time system of Indonesian is not expressed by morphological inflections, but by the time words “sudah” and “telah” [25]. “Sudah” is an adverb of time, equivalent to “*yijing*” in Mandarin Chinese. One usage of “sudah” is to indicate an action’s completion or a situation’s occurrence, which must precede the verb. An example is shown in (8).

Indonesian:  Ayah   sudah   memuji  kakak   dan    adik Chinese:        爸爸   已经     表扬     哥哥    和     姐姐 English word:   Dad    already  praise   brother  and  elder sister English: Dad has praised her brother and elder sister(8)

As shown in the coding of time information, Mandarin Chinese and Thai can use aspect markers and their combination with time adverbs, while Indonesian has no language item corresponding to the Chinese aspect marker “*le*”. In this study, we selected Mandarin Chinese learners with Thai and Indonesian L1 backgrounds as participants. The similarities and differences in the time coding approaches of Chinese, Thai, and Indonesian enable us to investigate the effect of the correspondence between L2 learners’ L1 and L2 on L2 processing. The meanings of time adverbs (Chinese, Thai, and Indonesian) and aspect markers (Chinese and Thai) are shown in Table 1.

### 1.3. The Present Study

The present study used ERP to investigate the processing mechanism of aspect agreement in Mandarin Chinese by L2 Chinese learners with different L1 backgrounds. We manipulated the agreement relation between the time adverbs “*yijing*” “*zhengzai*” and aspect maker “*le*”. Our findings will shed light on the neurocognitive mechanism of L1/L2 aspect processing.

According to the previous relevant research, we had the following predictions for our experiments. First, given that Mandarin Chinese aspect markers are morphologically marked and are attached after the verbs [8,9,10], both LAN and P600 could be evoked by aspect violation in native speakers. Second, as for the cross-language comparisons, there are no grammatical items corresponding to Chinese “*le*” in Indonesian. Moreover, although “แล้ว” in Thai can be used to express the completion of an action, it does not fully correspond to “*le*” in Chinese. Following the Shallow Structure Hypothesis, the second language learners may not reach the automatic processing level of processing complex or abstract syntactic structure as native speakers, and they may only reach the processing level similar to native speakers in the processing of local dependency structure. Specifically, according to the Competition Model [26,27,28] and the Shallow Structure Hypothesis [29], second language learners with Thai background may not induce typical LAN and P600, and second language learners with Indonesian background may fail to induce LAN or P600. Thus, there could be different ERP patterns among L2 learners with Thai backgrounds and Indonesian backgrounds, and these patterns may differ from those of Mandarin Chinese native speakers.

## 2. Materials and Methods

### 2.1. Experiment Design

The present study adopts a 2 (condition: aspect correct and violation) × 3 (participant group: Chinese native group, Indonesian group and Thai group) mixed design.

### 2.2. Participants

There were a total of 69 participants in this experiment. The experiment groups included L2 Chinese learners with Indonesian backgrounds (the “Indonesian group”) and L2 Chinese learners with Thai backgrounds (the “Thai group”). There were 23 people in each group, and in subsequent data processing, the data from three subjects in each group were removed because of too many artifacts. Therefore, data from 20 subjects were included in the final analysis in each group. The mean average and standard deviation of the native group, Indonesian group, and Thai group were, respectively, 19.5/1.1, 23.2/2.4, and 20.3/1.5. None of the L2 participants were ethnic Chinese, and they were college students who had all passed HSK-5. They lived in Thailand and Indonesia, respectively, before they went to college. They had lived in China for more than three years and were there when they took part in this experiment. None of them had learned Chinese before the age of 12. Twenty Chinese native speakers served as the control group. All participants were right-handed and healthy, and had no nervous system disease, or history of brain injury and/or dyslexia. All had normal vision (or normal vision after correction). We obtained informed consent from each participant before the experiment. After the experiment, all participants were given a monetary honorarium. This study was approved by the ethics committee at the Huaqiao University School of Medicine (ethics code: M2021014).

Although the L2 participants in the study are highly proficient in Mandarin (e.g., passed the HSK-5), we still adopted the self-assessment questionnaire of communicative language ability proposed by Bachman and Palmer [30] to evaluate their Chinese proficiency in a unified standard. The questions probed in the questionnaire include the perceived difficulty in the following three aspects: Grammatical competence (morphology and syntax), Pragmatic competence (vocabulary, cohesion, and organization) and Sociolinguistic competence (register, nativeness, and nonliteral language). Bachman and Palmer [30] proved that this test has high reliability and validity. According to the analysis of ERP data, the data of 3 subjects in each group were excluded, so we only reported the data of 20 effective subjects in each L2 group. The self-evaluation results of the two groups of L2 participants under each dimension were analyzed by the mixed effect model, as shown in Table 2. The results show that there is no significant difference in Chinese proficiency between the two L2 groups.

### 2.3. Materials

To explore the issue of aspect processing, we adopted a violation paradigm, which has been commonly used in sentence processing. Specifically, this study included two types of sentences: correct sentences and aspect violation sentences (see Table 3 for experiment examples). Aspect violation sentences were sentences that did not conform to syntactic rules. For example, in “*Baba yijing/zhengzai biaoyang le gege he jiejie*”, the time of the adverb “*yijing*” points to the past, the aspect marker “*le*” is attached to the verb “*biaoyang*” to indicate completion, and the combination of “*yijing*” and “*le*” provides a reference for past time information. If “*zhengzai*” (indicating that the action is in progress) and “*le*” (indicating that the action has been completed) are combined, a violation will occur in the time agreement.

Since the purpose of this study was to investigate the neural mechanism of second language grammatical processing, we controlled the familiarity with the experiment materials. All the experiment materials were taken from HSK grade A vocabulary. The experiment sentences consisted of seven segments. Three L2 Chinese teachers with doctoral degrees evaluated the sentence acceptability, and we also invited three L2 Chinese learners (whose Chinese levels are equivalent to the L2 participants in the ERP experiment) to judge the experiment material familiarity. Finally, a total of 90 pairs of key materials (a pair consists of one correct sentence and one violation sentence, and they are identical except the adverbs of time) were enrolled in the formal experiment, as shown in Table 3.

Ninety pairs of experiment materials were divided into two list according to a Latin-square procedure to ensure that the sentences in one pair do not appear in the same list. During the experiment, each participant received only one list. In this way, each participant had to read a total of 90 key sentences (45 sentences per condition). In order to balance the participants’ “yes” and “no” responses in the ERP experiment, the participants also read 120 fillers in addition to the key sentences. Among these fillers, there were 60 correct sentences in the form of “S + V + O” (30 contained “*zhengzai*” and 30 contained “*yijing* + “*le*” (at the end of the sentence)), and 60 sentences had syntactic errors. All 210 sentences in each list were pseudo-randomized, with the restriction that no more than three consecutive sentences with the same conditions. The experiment materials were compiled in E-prime 3.0.

### 2.4. Procedure

We conducted the experiment in an electromagnetic shielded laboratory. Participants sat about 80 cm away from a computer screen. Before the experiment, the participants had to read the instructions carefully to ensure that they understood the experiment’s requirements. After that, the participants began to complete 10 exercises, which could be repeated until they were proficient in the experiment process. In the formal experiment, we divided each sentence into seven segments (according to the words in the example sentence, a word is a chunk) and presented them segment-by-segment in the center of the computer screen. Each segment was presented in white font in the center of the gray screen. At the beginning of the experiment, the shape of a cross appeared on the screen for 800 ms, then an empty screen for 600 ms, then each segment was presented and lasted for 600 ms. There was a 200 ms interval between segments, the last segment appeared with a period, and a 600 ms empty screen appeared after the sentence. Then, three question marks appeared in the center of the screen for 3000 ms and at this time, the participants needed to press a key to determine whether the sentence was acceptable. If the answer was YES, they had to press “F” with their left hand, and if the answer was NO, they had to press “J” with their right hand. The assignment of left/right hand to yes/no response was counterbalanced across participants.

### 2.5. Data Preprocessing and Analysis

The NeuroScan ERP system (with 64 electrodes, the international 10/20 system) was used to record EEG activity. The vertical electrooculogram (VEOG) was installed on the upper and lower sides of the left eye, and the horizontal electrooculogram (HEOG) from electrodes was placed on the outside of both eyes. Electrode impedances were kept below 5 kΩ. EEG signals were filtered using a bandpass of 0.016–700 Hz, and digitized at a sampling rate of 500 Hz. The EEG was referenced online to the left mastoid and re-referenced offline to the algebraic average activity measured in the left and right mastoids. EEG activity was filtered offline with a bandpass zero phase shift filter (high cutoff: 30 Hz, low cutoff: 0.05 Hz, 12 dB/oct). The ERP epoch was extracted for the critical aspect marker le for each critical sentence, with a pre-stimulus baseline of 200 ms and the ERP response to the critical word for 1000 ms. Trials with EEG maximal amplitude exceeding ±70 µV or with incorrect responses were eliminated from data analysis, resulting in 84.92% artifact-free trials for native speakers and 71.07% artifact-free trials for Indonesian speakers, and 73.63% artifact-free trials for Thai speakers. According to the above criteria, we eliminated three participants from each of the three groups due to excessive artifacts, and 20 participants in each group were included in the final statistical analysis. EEG activity was smoothed with a bandpass zero phase shift filter (high cutoff: 8 Hz, low cutoff: 0.05 Hz, 12 dB/oct) which was used only for displaying and not for statistics. ERPs were computed for each participant.

The ERP components we were interested in are LAN and P600. LAN is negativity distributed in the left anterior position of the scalp (known as the left anterior negativity). It is induced by morphological abnormalities and is correlated with morphosyntactic agreement processing [31,32]. P600 is a late positivity, most prevalent in the posterior part of the brain, and the peak appears at about 500–900 ms. It is usually sensitive to morphological and syntactic complexity [33].

Based upon visual inspection and previous studies (e.g., Friederici [31]; Hagoort et al. [32]; Neville [33]; Friederici [34]; Hahne and Friederici [35]; Mancini et al. [36]; Tanner and VanHell [37]; Beatty-Martínez et al. [38]), linear mixed models (LMMs) were conducted on ERP amplitudes in the selected time windows (100–300 ms for ELAN; 300–500 ms for LAN; 600–800 ms for native speakers and 450–900 ms for both L2 learners for the late positivity; see Section 3), with condition (aspect violation vs. correct sentence) and topographical factors as within-participant variables. We analyzed the average amplitude from the midline and bilateral parts of the scalp. For the midline analysis, the topographical factor was an electrode with three levels: anterior (FZ), central (CZ), and posterior (PZ). For the lateral analysis, the topographic factors were region (three levels: anterior vs. central vs. posterior) and hemisphere (two levels: left vs. right). The region and hemisphere were crossed to form six regions of interest: left frontal (F3, F5, F7, FC3, FC5, FT7), left central (C3, C5, T7, CP3, CP5, TP7), left parietal (P3, P5, P7, PO3, PO7, O1), right frontal (F4, F6, F8, FC4, FC6, FT8), right central (C4, C6, T8, CP4, CP6, TP8) and right parietal (P4, P6, P8, PO4, PO8, O2).

## 3. Results

In this study, linear mixed-effect regression models (LMMs) were constructed to simultaneously model the variance associated with each subject and each item using the lme4 package (Bates et al. [39]) in R (version 4.1.1).

We use the AICc () function of MuMIn package and anova () function to compare the models and the model selection procedures.

M.01 <-lmer (Avgamp~Point*Condition + (1|subject), data = x)/M.01 <-lmer (Avgamp~Hemi*Area*Condition + (1|subject), data = x)

M.02 <- lmer (Avgamp~1 + Point*Condition+(1|subject), data = x)/M.02 <-lmer (Avgamp~1 + Hemi*Area*Condition + (1|subject), data = x)

MuMIn:AICc (M.01)

MuMIn:AICc (M.02)

anova (M.01, M.02, refit = FALSE)

The running results told us that *p* < 0.001 in model M.01 and *p* = 1.000 in model M.02, which indicated that “Point” and “Condition”/“Hemi”, “Area”, and “Condition” present an interaction effect, so the fitting result of model M.01 was better.

The finally fitted model in the midline analysis included two factors: Mixed model = M.01<-lmer (Avgamp~Point*Condition + (1|subject), data = x), where Point represents the electrode point, Condition represents the condition, Avgamp represents the average amplitude. The finally fitted model in the lateral analysis included three factors: Mixed model = M.01<-lmer (Avgamp~Hemi*Area*Condition + (1|subject), data = x), where Hemi represents the hemisphere, Area represents the region, Condition represents the condition and Avgamp represents the average amplitude. We analyzed their main effects and interaction with the Anova() function. At the same time, we carried out the post hoc analysis with the glht() function, and we reported the *p*-values.

### 3.1. Behavioral Performance

Table 4 shows the average correct rates for participants in the three groups. The mixed-effects modeling analysis on the participants’ accuracy showed that the main effect of group was significant (χ^2^(2) = 188.720, *p* < 0.001), and the post hoc comparison suggested that the accuracy of the native group was significantly higher than that of the Thai group (β = −0.196, SE = 0.014, z = −13.710, *p* < 0.001); the accuracy of the native group was significantly higher than that of the Indonesian group (β = −0.109, SE = 0.014, z = −7.612, *p* < 0.001); the accuracy of the Thai group was significantly higher than that of the Indonesian group (β = −0.087, SE = 0.014, z = −6.098, *p* < 0.001). Follow-up analysis showed that for any of the three groups, the behavioral accuracy of the correct sentence was significantly higher than that of the violation sentence (ps < 0.001), suggesting that both native speakers and second language learners can distinguish these two types of sentences.

Table 4 also shows participants in three groups’ average reaction time. The mixed-effects modeling analysis on the participants’ reaction time showed that the main effect of group was significant (χ^2^(2) = 100.829, *p* < 0.001), and the post hoc comparison suggested that the average reaction time of the native group was significantly shorter than that of the Thai group (β = 0.750, SE = 1.090, z = 5.446, *p* < 0.001), as well as the Indonesian group (β = 0.560, SE = 1.090, z = −3.497, *p* = 0.001); the differences in the reaction time between the Thai group and the Indonesian group were not significant (β = 0.190, SE = 1.090, z = 1.949, *p* = 0.125).

For native speakers, follow-up analysis showed that the response time for the aspect violation sentences were longer than the correct sentences (β = 0.183, SE = 0.765, z = −2.183, *p* = 0.029), whereas there was no difference between the aspect violation sentences and the correct sentences in Indonesian group (χ^2^(1) = 0.403, *p* = 0.526) or the Thai group (χ^2^(1) = 1.892, *p* = 0.169).

### 3.2. ERP Data

The present study focuses on the ERP patterns of the different conditions (aspect correct and violation) for each group, so we will report them separately below.

#### 3.2.1. The Native Group

**Figure 1:**
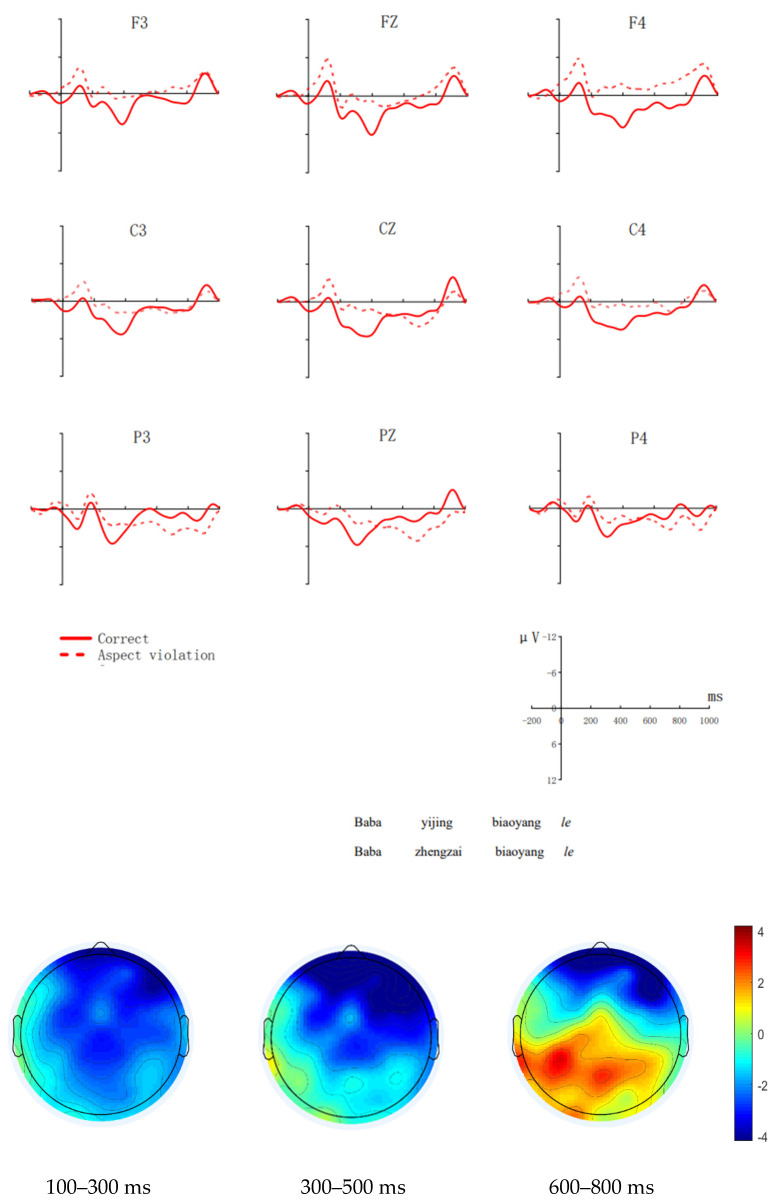
Grand average (N = 20, with 20 valid samples) ERPs of the native speaker group at 9 exemplar electrodes time-locked to the onset (0 ms) of the aspect marker “*le*” as a function of aspect agreement. The *x*-axis represents the duration, and each hash mark represents 200 ms. The *y*-axis represents the voltage, which ranged from −12 to +12 µV. Negativity is plotted upward. ELAN, LAN-like component and P600 effect’s scalp distribution are depicted in the topographic maps. The topographical voltage map represents the difference waves effects (the aspect violation condition minus the correct condition) at 100–300 ms, 300–500 ms, and 600–800 ms, respectively.

Figure 1 shows waveforms from aspect violation sentences and correct sentences, and scalp distributions of aspect violation processing in the ELAN, LAN, and P600 time window. Our experimental paradigm is “violation paradigm”, referring to the experimental paradigm of Zhang and Zhang [15] and Qiu and Zhou [10]. Within this paradigm, the difference between the two conditions appeared before and after 0 ms with the naked eye, when the critical word had not yet appeared or appeared for a very short time. In this paradigm, the correct sentence is exactly the same as the aspect violation sentence except for the time adverb (See Table 3). The difference caused by different time adverbs (“*yijing*” and “*zhengzai*”) and the elicitation of subjects’ expectations for these two time adverbs may have continued up to the baseline of the keyword, so the difference between the two conditions appeared before and after 0 ms, when the critical word had not yet appeared or appeared for a very short time. This was also the case in their studies (Zhang & Zhang, 2008; Qiu and Zhou, 2012). Although the difference is visible to the naked eye, there is no statistical difference between the two conditions appearing at the baseline (−200–0 ms) and before and after 0 ms (−200–60 ms).

- Midline

We analyzed the average amplitude of the critical word in the 100–300 ms time window. The model comparison with LMMs the processing of Chinese aspect violations of showed a significant main effect of condition (χ^2^(1) = 4.071, *p* = 0.039); post hoc comparison suggested that the ERP responses evoked by aspect violation sentences were larger than those evoked by correct sentences (β = −2.169, SE = 0.659, z = −4.257, *p* = 0.037). The interaction effect between electrodes and condition was not significant (χ^2^(2) = 4.306, *p* = 0.116).

We analyzed the average amplitude of the critical word in the 300–500 ms time window. The model comparison failed to show any interesting main effects or interactions. In the 600–800 ms window, the model comparison showed a significant main effect of condition (χ^2^(1) = 6.617, *p* = 0.020), and post hoc comparison suggested that the ERP responses evoked by aspect violation sentences were larger than those evoked by correct sentences (β = 1.738, SE = 1.227, z = 3.362, *p* = 0.017). The interaction effect between electrodes and condition was marginally significant (χ^2^(2) = 4.783, *p* = 0.068), and the post hoc comparison suggested that the ERP responses evoked by aspect violation sentences were larger than those evoked by correct sentences at PZ (β = 2.038, SE = 1.227, z = 2.362, *p* = 0.017). There was no significant difference in the ERP responses induced by aspect violation sentences and correct sentences at CZ and FZ.

- Lateral

We analyzed the average amplitude of the critical word in the 100–300 ms time window. The model comparison with LMMs showed a significant two-way interaction between condition and hemisphere (χ^2^(1) = 9.040, *p* = 0.003), and the post hoc comparison suggested that the ERP responses evoked by aspect violation sentences were larger than those evoked by correct sentences at the left hemisphere (β = −0.817, SE = 0.392, z = −2.083, *p* = 0.037). Other interactions were not significant.

At 300–500 ms, the model comparison showed an insignificant main effect of condition (χ^2^(1) = 0.058, *p* = 0.810). However, the interaction between condition and hemisphere was significant (χ^2^(1) = 6.412, *p* = 0.011), and post hoc comparison suggested that the difference of the ERP responses evoked by aspect violation sentences and correct sentences was marginally significant at the left hemisphere (β = 1.163, SE = 0.602, z = 1.934, *p* = 0.053); the interaction between condition and region was significant (χ^2^(2) = 6.501, *p* = 0.047), and post hoc comparison suggested that ERP responses evoked by aspect violation sentences were larger than those evoked by correct sentences at the frontal region (β = 2.749, SE = 0.762, z = 2.983, *p* = 0.024). The three-way interaction was not significant (χ^2^(2) = 0.822, *p* = 0.663).

In the 600–800 ms time window, the model comparison showed a significant main effect of condition (χ^2^(1) = 19.020, *p* = 0.049), with larger ERP responses for aspect violation sentences than for correct sentences (β = 2.243, SE = 0.541, z = 4.363, *p* = 0.043). Moreover, the interaction between condition and hemisphere was significant (χ^2^(1) = 12.038, *p* = 0.024), and post hoc comparison suggested that the ERP responses evoked by aspect violation sentences were larger than those evoked by correct sentences at the right hemisphere (β = 3.222, SE = 0.717, z = 4.496, *p* < 0.001); the interaction between condition and region was significant (χ^2^(2) = 23.936, *p* = 0.016), and post hoc comparison suggested that ERP responses evoked by aspect violation sentences were larger than those evoked by correct sentences at the parietal region, (β = 3.669, SE = 0.900, z = 4.077, *p* < 0.001). The three-way interaction was not significant (χ^2^(2) = 0.649, *p* = 0.723).

#### 3.2.2. The Indonesian Group

**Figure 2:**
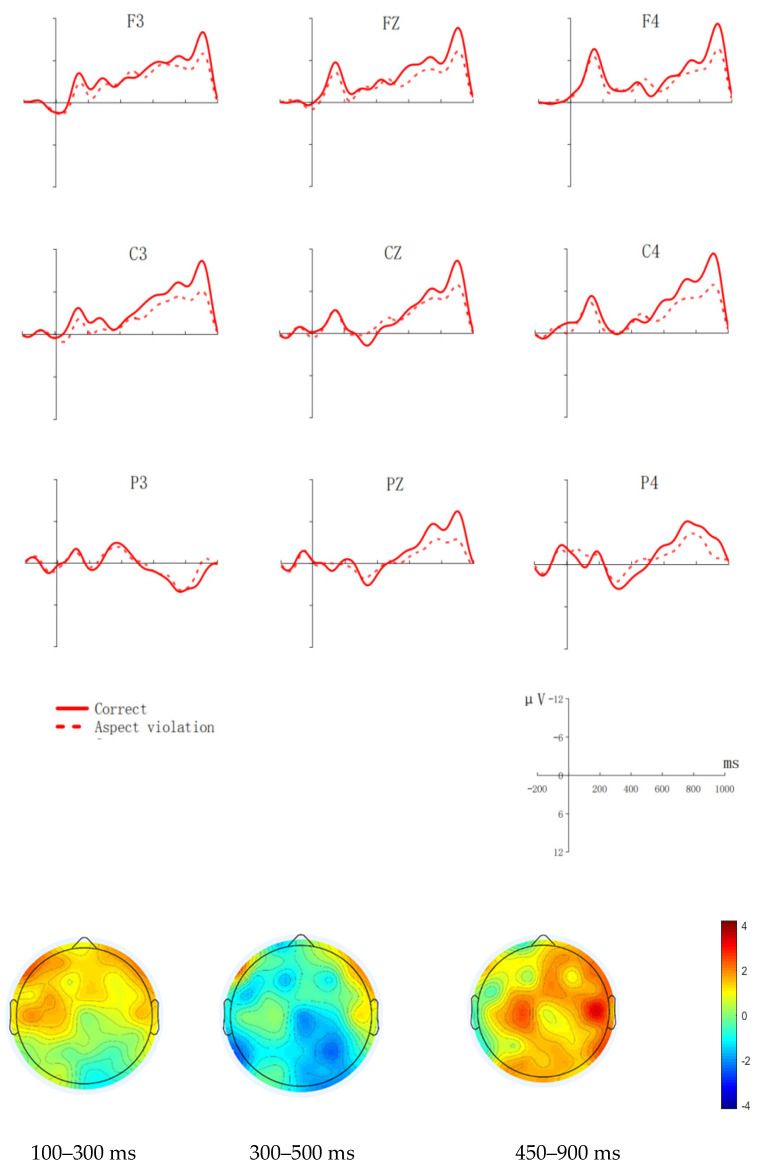
Grand average (N = 20, with 20 valid samples) ERPs of the Indonesia group at 9 exemplar electrodes time-locked to the onset (0 ms) of the aspect marker “*le*” as a function of aspect agreement. The *x*-axis represents the duration, and each hash mark represents 200 ms. The *y*-axis represents the voltage, which ranged from −12 to +12µV. Negativity is plotted upward. The topographical voltage map represents the difference waves effects (the aspect violation condition minus the correct condition) at 100–300 ms, 300–500 ms, and 450–900 ms, respectively.

Figure 2 shows the waveforms of aspect violation sentences and correct sentences, and the scalp distributions of aspect violation processing.

- Midline

We analyzed the average amplitude of the critical word in the 100–300 ms time window. The model comparison showed that condition’s main effect was not significant (χ^2^(1) = 0.086, *p* = 0.769); The interaction between electrode and condition was not significant either (χ^2^(2) = 0.498, *p* = 0.780).

We analyzed the average amplitude of the critical word in the 300–500 ms time window. The model comparison failed to show any interesting main effects or interactions. We also analyzed the average amplitude of the critical word in the 450–900 ms time window. The model comparison showed that the condition’s main effect was not significant (χ^2^(1) = 2.213, *p* = 0.137). The interaction effect between the electrode and the condition was not significant either (χ^2^(2) = 0.328, *p* = 0.849).

- Lateral

In the 100–300 ms time window, the model comparison failed to show any interesting main effects or interactions.

At 300–500 ms, the model comparison showed an insignificant main effect of condition (χ^2^(1) = 0.198, *p* = 0.657). However, the interaction between condition and hemisphere was significant (χ^2^(1) = 6.007, *p* = 0.035), and post hoc comparison suggested that the ERP responses evoked by aspect violation sentences were larger than those evoked by correct sentences at the right hemisphere (β = 2.212, SE = 0.845, z = 2.251, *p* = 0.042). 

In the 450–900 ms time window, the model comparison showed that although the condition’s main effect was not significant (χ^2^(1) = 0.018, *p* = 0.893), there was a significant two-way interaction between condition and hemisphere (χ^2^(1) = 4.062, *p* = 0.044). The post hoc comparison suggested that the ERP responses evoked by aspect violation sentences was more positive than those evoked by correct sentences at the right hemisphere (β = −0.914, SE = 0.548, z = −1.667, *p* = 0.065). No other interactions were significant.

#### 3.2.3. The Thai Group

**Figure 3:**
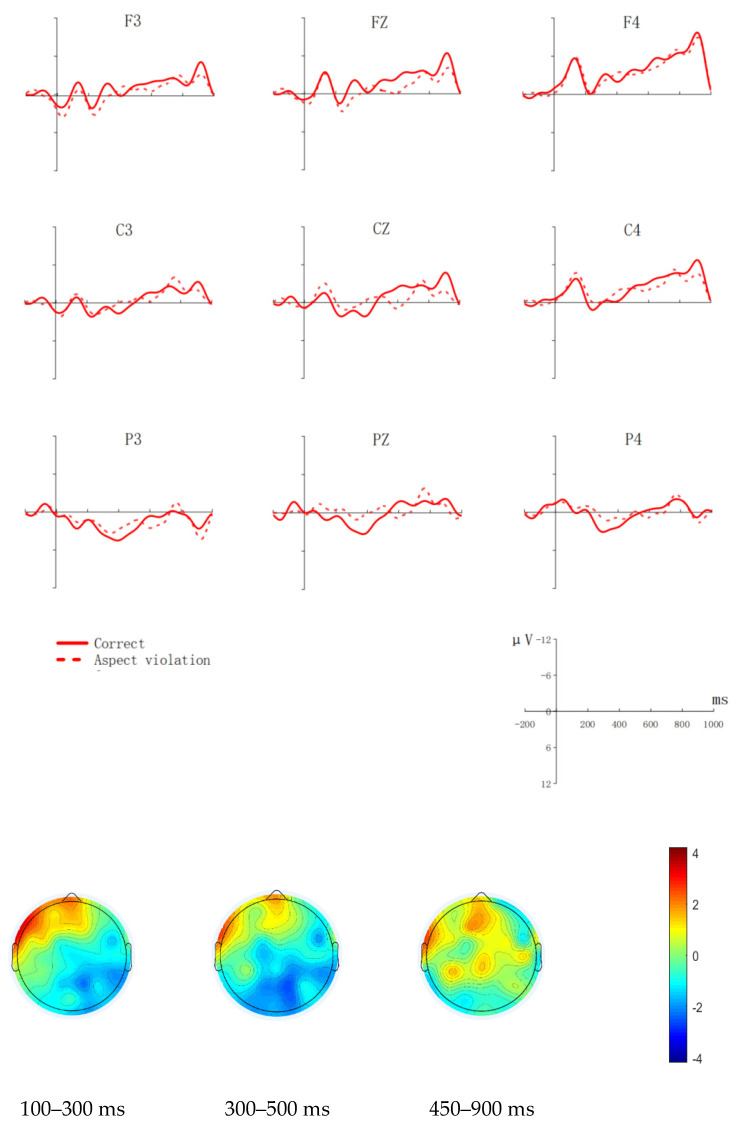
Grand average (N = 20, with 20 valid samples) ERPs of the Thailand group at 9 exemplar electrodes time-locked to the onset (0 ms) of the aspect marker “*le*” as a function of aspect agreement. The *x*-axis represents the duration, and each hash mark represents 200 ms. The *y*-axis represents the voltage, which ranged from −12 to +12 µV. Negativity is plotted upward. The topographical voltage map represents the difference waves effects (the aspect violation condition minus the correct condition) at 100–300 ms, 300–500 ms, and 450–900 ms, respectively.

Figure 3 shows waveforms of aspect violation sentences and correct sentences and a scalp distribution of aspect violation processing in the P600 time window.

- Midline

We analyzed the average amplitude of the critical word in the 100–300 ms time window. The model comparison failed to show any interesting main effects or interactions.

In the 300–500 ms window, the model comparison showed a significant main effect of condition (χ^2^(1) = 12.242, *p* < 0.001), with larger ERP responses for aspect violation sentences than for correct sentences (β = −2.695, SE = 0.770, z = −3.503, *p* < 0.001). The interaction effect between electrodes and condition was not significant (χ^2^(2) = 0.201, *p* = 0.904). In the 450–900 ms time window, there was a marginally significant two-way interaction between condition and electrode (χ^2^(2) = 4.847, *p* = 0.089), and the post hoc comparison suggested that the ERP responses evoked by aspect violation sentences were larger than those evoked by correct sentences at CZ (β = 2.812, SE = 1.322, z = 2.370, *p* = 0.025) and at FZ (β = 3.234, SE = 0.873, z = 3.325, *p* = 0.018).

- Lateral

The model comparison failed to show any interesting main effects or interactions in the 100–300 ms time window.

At 300–500 ms, the model comparison showed a marginally significant main effect of condition (χ^2^(1) = 3.012, *p* = 0.083). Moreover, the interaction between condition and hemisphere was significant (χ^2^(1) = 7.086, *p* = 0.030), and post hoc comparison suggested that the ERP responses evoked by aspect violation sentences were larger than those evoked by correct sentences at the right hemisphere (β = −2.111, SE = 0.666, z = −3.171, *p* = 0.008). 

In the time window of 450–900 ms, there was a significant two-way interaction between condition and region (χ^2^(2) = 6.749, *p* = 0.034), and the post hoc comparison suggested that the ERP responses evoked by aspect violation sentences were more positive than that evoked by correct sentences in the frontal regions, (β = 2.278, SE = 0.533, z = 3.398, *p* = 0.016). No other interesting effects were significant.

## 4. Discussion

In this study, we found that the aspect violation in Mandarin Chinese induced an obvious triphasic ELAN-LAN-P600 effect in the native group, but only 300–500 ms negativity widely distributed in the right hemisphere (referred to as an RN) and P600-like effect was elicited in the Indonesian group and Thai group. The cross-group comparison showed that the P600 modulation was smaller in the second language group than in the native group. In the comparison between the two second language group, although there were differences in behavioral data, there was no difference in ERP patterns.

### 4.1. The Native Speaker Group

In the present study, the negativity in the 100–300 ms time window, which was evoked by aspect violation, was most pronounced in the left hemisphere and frontal region. Given its functional significance and scalp distribution, this negativity is regarded as ELAN. ELAN reflects the automatic construction of syntactic structure, such as the recognition of parts of speech or categories [34,35]. The Mandarin Chinese particle “*le*” has been traditionally treated as an aspectual particle, expressing the completion of an action/event. This is consistent with the statement that Mandarin Chinese aspect markers are morphologically similar to the inflection suffixes of most Indo-European languages’ verbs, and attached to the verb [8,9]. Therefore, the ELAN in this study indicated that the native group constructed the syntactic structure automatically, namely, they identified the aspect violation.

In the 300–500 ms time window, according to the statistical results, waveforms, and topographic maps, we found that native speakers had a LAN-like component, which was more obvious in the left hemisphere and anterior area. According to previous literature (e.g., Mancini et al. [36]; Tanner and VanHell [37]; Beatty-Martínez et al. [38]), the LAN appearing in the 300–500 ms window is related to morphosyntax processing, therefore, it may indicate that the native group had a morphosyntactic information integration processing for “*le*” in the aspect violation sentence.

In the late time window (450–900 ms), aspect violation in the native group elicited a larger positive effect (P600), which was most pronounced in the parietal regions. This is consistent with Zhang and Zhang [15]. Previous studies showed that P600 reflects syntactic repair or the monitoring and solving of conflicts caused by aspect violation [7,10,40], we thus believe that the occurrence of P600 could be mainly driven by the efforts to repair/re-interpret the aspect disagreement. For native speakers, the aspect violation elicited a LAN-P600 biphasic effect, which is consistent with previous studies concerning morphological processing (e.g., Steinhauer and Ullman [12]). Steinhauer and Ullman [12], for example, have found a LAN-P600 during processing inflectional morphology involving regular and irregular past-tense violations in English.

### 4.2. The Second Language Group

In the 100–300 ms time window, we failed to find any effect, neither for the Indonesian group or the Thai group. The absence of the ELAN effect in these two groups suggests that non-native speakers of Mandarin Chinese have difficulty in detecting the grammatical disagreement between the aspect marker “*le*” and the time adverbs automatically (“*zhengzai*”, the action is in progress).

In the 300–500 ms time window, according to the statistical results, waveforms and topographic maps, we found that the Indonesian group and the Thai group had a negativity in the right hemisphere. Unlike the native group, L2 learners prefer to use the right hemisphere to process the morphosyntax “*le*” in Mandarin Chinese. This finding, which has also been reported in previous studies [41,42,43], suggests that learners who start learning a second language after adolescence tend to use the right hemisphere to process the second language.

In the 450–900 ms time window, the Indonesian group and the Thai group showed a P600-like effect in response to aspect violation which may indicate that they have some degree of sensitivity to aspect violation and try to repair/re-interpret the conflicts. However, the lack of ELAN and LAN for L2 Chinese learners suggests that L2 Chinese learners with both Indonesian and Thai backgrounds cannot reach the same level of automatic processing as Chinese native speakers in Chinese aspect processing.

There are two different types of “*le*” in Mandarin Chinese, one is a perfective marker which comes right after the verb, and the other is a modal particle which comes at the end of the sentence. Therefore, the usage of “*le*” is very complicated in Mandarin Chinese. The acquisition of perfective “*le”* is usually difficult for many Chinese L2 learners because their native language does not distinguish this function. Indonesian, for example, has no corresponding grammatical items. Compared to Indonesian, Thai has corresponding grammatical items, but it cannot fully correspond to it in meaning and usage. Duff and Li [44] examined both oral and written works of L2 Chinese college students, and they found that L2 learners, particularly those with lower proficiency levels, tend to undersupply “*le”* in oral utterance, suggesting that L2 learners have difficulty in acquiring the perfective aspect marker “*le*”.

There are no grammatical items corresponding to “*le*” in Indonesian learners’ L1, therefore, participants with Indonesian background may directly use the processing strategies in their L1 to process “*le*”. The Indonesian group, therefore, did not show an ELAN-LAN-P600 triphasic effect on Chinese aspect violations, although an RN in the 300–500 ms and widely distributed late positive effect were observed in the 450–900 ms window. In fact, the existing evidence demonstrates that even proficient L2 learners differ from native speakers when processing syntactic features that are absent in their native language [45]. Qiu and Zhou [10] found that the violation of time adverbs elicited the N400-P600 effect. Therefore, if the processing of the Indonesian time adverb “sudah” can be compared with that of the Chinese past time adverb “*yijing*”, then L1 may affect the aspect marker processing of L2 Chinese learners with Indonesian backgrounds, resulting in ERP components involving lexical semantic processing. However, in this study, the Indonesian group did not elicit the typical N400 effect, but induced an RN-P600-like effect. This indicated that the Indonesian native speakers may depend on a neurocognitive mechanism which differs from the Chinese native speakers in dealing with the Mandarin Chinese aspect violation processing. However, it was unclear what kind of processing mechanism was involved when processing the Chinese aspect information, and whether it is syntactically or lexically driven.

According to the unified competition model, Thai learners have grammatical items corresponding to “*le*” in their L1, but they cannot fully correspond to Mandarin Chinese in meaning and function, resulting in competition and negative transfer. The Thai group did not have an ELAN-LAN-P600 triphasic effect on Chinese aspect violations, but there was an RN-P600-like effect. Park [46] studied the cross-linguistic influence in morphological syntactic processing and found that it was difficult for participants to integrate L2 syntactic structures that were not similar to L1. In Thai, the meaning and function of “ แล้ว” and Chinese aspect mark “*le*” are not exactly equivalent. L2 Chinese learners with Thai backgrounds detected aspect mismatch when processing “*le*” and tried to repair it, but it was difficult to fully integrate relevant syntactic information, therefore, an RN-P600-like effect was elicited.

According to the shallow structure hypothesis [29], unlike native speakers, L2 learners cannot reach the automatic processing level of processing complex hierarchical structures or abstract syntactic structures (such as syntactic ambiguity). Different levels of second language syntactic processing have been widely discussed [47,48,49]. This research showed that high-level L2 learners’ processing patterns were similar to those of native speakers, while there were differences between low-level L2 learners and native speakers. This suggests that the underlying mechanism was modulated by L2 proficiency. Relative to native speakers, low-level L2 learners tend to rely on shallow lexicons or probability to process language items, while high-level L2 learners depend on simple syntactic points [50]. Among beginners, both grammar and lexicon processing tend to rely on declarative memory systems, but with the continuous improvement in L2 proficiency, grammar processing gradually begins to rely on procedural memory systems [51]. Although all the participants in this experiment were high-level L2 learners (HSK5 served as the basis to define their Mandarin Chinese levels), the post-experiment interview showed that L2 learners’ Mandarin Chinese proficiency had not approached the level of native speakers. The absence of ELAN in the 100–300 ms time window, LAN in the 300–500 ms time window, and the typical P600 in the 450–900 ms time window indicates that L2 learners may prefer to rely on the declarative memory system and shallow language knowledge processing, and may adopt processing of neurocognitive mechanisms different to that of native speakers in the processing of Mandarin Chinese aspect violation.

There was no difference in processing data between the two L2 groups, but there was a difference in accuracy. Thai is more similar to Chinese in terms of aspect coding than Indonesian, which is the probable reason for their different accuracy rates. Accuracy rate belongs to behavior data, which may reflect the subjects’ mastery of language knowledge, while ERP data belong to implicit data, which is more sensitive and can reflect the real-time language processing ability of the subjects [16]. Although Thai native speakers had higher accuracy than Indonesian native speakers due to the positive transfer of their native language, they were still unable to process aspect markers as Chinese native speakers did because of the complexity of the usage of “*le*”. Previous studies found that high-level L2 learners present ERP effects similar to those of L1 speakers when processing local dependency structures (e.g., subject–predicate agreement, suffix change, and gender and number agreement) [52,53]. However, when processing the complex structure such as the syntactic ambiguity and the filler-gap dependencies, their performance would differ from L1 speakers [54,55,56,57,58], suggesting that L2 learners use the same mechanism as that of the native speaker only when processing local structures. The Mandarin Chinese aspect marker “*le*” is not only a crucial grammatical item in teaching Chinese as a foreign language, but also a challenging grammatical item [59]. It brings a lot of difficulties to the Chinese reading process, especially for second language learners of Chinese. Therefore, the processing data tend to indicate that there may be no essential difference in the processing mechanism of Mandarin Chinese aspect marker between the two groups of second language speakers.

### 4.3. The Mechanism of the Difference Found among the Three Groups

ELAN-LAN-P600 pattern appeared in the Chinese native speaker group, which indicates that Chinese native speakers can automatically identify the aspect violation, integrate morphosyntactic information, and reinterpret the mismatch between the aspect marker “*le*” and the time adverb.

More specifically, ELAN reflects the automatic construction of syntactic structure, such as the recognition of parts of speech or categories [34,35], LAN reflects more automated detection of morphological syntactic agreement errors [31], and P600 reflects the effort to repair the grammatical mistake and integrate it into a sentence representation. The lack of ELAN and LAN and the negative effect in the right hemisphere in 300–500 ms suggest that the Thai and Indonesian groups cannot reach the level of automatic processing for Chinese aspect violation although they are highly proficient learners of Chinese (passed HSK5); it also suggests that the neural mechanism underlying of Chinese aspect agreement processing is different for the native speakers and non-native speakers. As for the RN and late positivity, further studies are needed to explore how second language learners’ native language background influences their real-time processing of Chinese grammatical information.

There may be some limitations in our discussion. As for the Chinese proficiency of the two L2 groups, although HSK level and the self-rated Chinese proficiency were used to make sure that there was no significant difference between the two L2 groups, the results of the off-line test on acceptability judgment (accuracy) suggest some difference between the two groups, which could be potential confounding factors that should be controlled in further studies.

## 5. Conclusions

In this study, we investigated how native Chinese speakers and L2 Chinese learners with different L1 backgrounds process aspect violation in Mandarin Chinese. We involved three groups of participants: native Chinese speakers, L2 Chinese learners with Thai backgrounds, and L2 Chinese learners with Indonesian backgrounds. The experiment results showed that native speakers elicited an ELAN-LAN-P600 triphasic effect under the aspect violation condition, while L2 Chinese learners with different L1 backgrounds showed different ERP effects. L2 Chinese learners with Thai backgrounds and L2 Chinese learners with Indonesian backgrounds showed no ELAN and LAN under the condition of aspect violation, but an RN-P600-like effect. The lack of ELAN and LAN, and the negative effect in the right hemisphere in 300–500 ms in L2 groups reflect the failure of automatic processing of Chinese aspect markers, and the late positivity may reflect syntactic repair or the resolution of conflicts caused by aspect violations. Compared with native speakers, L2 Chinese learners adopt a cognitive neural mechanism which is different from that of native Chinese speakers.

## Figures and Tables

**Table 1:** Time adverbs (Chinese, Thai, and Indonesian) and aspect markers (Chinese and Thai).

	Chinese/Thai/Indonesian
Time Adverbs	已经 (yijing)/แล้ว/sudah: “已经 (yijing)/แล้ว/sudah” is an adverb, and it is placed before the verb to indicate that the time has passed or that the action, situation, and thing were completed before a certain time. 正在 (zhengzai)/กำลัง/sedang: “正在 (zhengzai)/กำลัง/sedang” is an adverb, and it is placed before the verb to indicate that the action is in progress.
Aspect markers	了 (le)/แล้ว: “了 (le)/แล้ว” is an aspect marker, and it is appended to the verb to indicate that the action has been completed. 着 (zhe)/กำลัง: “着 (zhe)/กำลัง” is an aspect marker, and it is appended to a verb to indicate that the action is in progress.

**Table 2:** Statistical results of L2 proficiency self-assessment between the Indonesian group and the Thai group.

Dimension	Mean of Indonesian Group	Mean of Thai Group	Chisq	Df	Pr (>Chisq)
Grammatical competence	2.871	2.893	0.379	1	0.538
Pragmatic competence	2.800	2.786	0.174	1	0.676
Sociolinguistic competence	2.643	2.607	0.798	1	0.371

Note: Chisq is Chi-square value; Df is degree of freedom; Pr is p-value.

**Table 3:** Examples of sentences in each condition.

Correct 表扬 了 哥哥 和 姐姐 Dad already praise *le* brother and elder sister Dad has already praised her brother and sister	爸爸 已经
	Baba yijing biaoyang *le* gege he jiejie
Aspect violation *爸爸 正在 表扬 了 哥哥 和 姐姐 Baba zhengzai biaoyang *le* gege he jiejie Dad is praising *le* brother and elder sister Dad is praising her brother and elder sister

**Table 4:** Average accuracy (ACC) rate and reaction time (RT) of different sentences in each group.

	Native Speakers Group		Indonesian Group		Thai Group	
	ACC (%)	RT (ms)	ACC (%)	RT (ms)	ACC (%)	RT (ms)
Correct	89.13/3.46	452.95/239.59	74.46/4.37	615.77/311.71	76.90/3.86	667.96/382.39
Aspect violation	80.71/5.21	504.21/226.20	67.67/6.25	634.64/297.48	70.35/4.72	723.24/432.25

Note: The number after “/” is SD.

## Data Availability

The data presented in this study are available on request from the corresponding author.
