# Correction: Hao et al. Processing Aspectual Agreement in a Language with Limited Morphological Inflection by Second Language Learners: An ERP Study of Mandarin Chinese. *Brain Sci.* 2022, *12*, 524

**DOI:** 10.3390/brainsci13081135

**Published:** 2023-07-28

**Authors:** Yuxin Hao, Xun Duan, Qiuyue Yan

**Affiliations:** 1 Institute of Chinese Language and Culture Education, Huaqiao University, Xiamen 361021, China; 2 College of Chinese Language and Culture, Huaqiao University, Xiamen 361021, China; 19014041006@stu.hqu.edu.cn (X.D.); 18014041031@stu.hqu.edu.cn (Q.Y.)

## Incorrect Affiliation

In the published publication [1], there was an error regarding the affiliation for Qiuyue Yan. **Affiliation number 3, “Suizhou No. 2 High School, Suizhou 441300, China”** need to be changed to his **Current Address**. The authors state that the scientific conclusions are unaffected. This correction was approved by the Academic Editor. The original publication has also been updated.
